# Please Read

**Published:** 1877-12-20

**Authors:** 


Please Read.—We will not send out renewal papers, but will take it for granted that all of our subscribers will renew their subscriptions; and, unless they notify us not to do so before the issue of our next number (January 20th), we will enter them on the list for 1878.

We hope that this notice will be borne in mind, and that every one of our subscribers will continue the Record, and will, without failure, remit his subscription promptly on the reception of the January number, and sooner if possible. Not only so, but that he will aid us in extending our circulation. Friends, we intend to give you a splendid volume for 1878.

